# Effect of Pre-Fixation Delay and Freezing on Mink Testicular Endpoints for Environmental Research

**DOI:** 10.1371/journal.pone.0125139

**Published:** 2015-05-01

**Authors:** Ellinor Spörndly-Nees, Elisabeth Ekstedt, Ulf Magnusson, Azadeh Fakhrzadeh, Cris L. Luengo Hendriks, Lena Holm

**Affiliations:** 1 Department of Anatomy, Physiology and Biochemistry, Faculty of Veterinary Medicine and Animal Science, Swedish University of Agricultural Science, Uppsala, Sweden; 2 Department of Clinical Sciences, Faculty of Veterinary Medicine and Animal Science, Swedish University of Agricultural Science, Uppsala, Sweden; 3 Department of Information Technology, Division of Visual Information and Interaction, Centre for Image Analysis, Uppsala University, Uppsala, Sweden; University of New South Wales, AUSTRALIA

## Abstract

There is growing interest in using wild animals to monitor the real-life cocktail effect of environmental chemicals on male reproduction. However, practical difficulties, such as long distances to the laboratory, generally prolong the time between euthanisation and specimen handling. For instance, tissue fixation is often performed on frozen material or on material where deterioration has started, which may affect tissue morphology. This study examined the effect of pre-fixation delay and freezing on mink testicular endpoints in order to determine robust endpoints in suboptimally handled specimens. Sexually mature farmed mink (n=30) selected at culling were divided into six groups and subjected to different time intervals between euthanisation and fixation or freezing: 0 hours (fixed immediately post mortem), 6 hours, 18 hours, 30 hours, 42 hours, or frozen 6 hours post mortem and thawed overnight. Unaffected endpoints when pre-fixation storage was extended to 30 hours included: area and diameter of the seminiferous tubules, length and weight of the testes, and acrosomes marked with Gata-4. Epithelial height, Sertoli cells marked with Gata-4 and cell morphology were affected endpoints after 6 hours of storage. Freezing the tissue prior to fixation severely altered cell morphology and reduced testicular weight, tubular diameter and area. Morphological changes seen after 6 hours included shredded germ cells and excess cytoplasm in seminiferous tubular lumen, chromatin rearrangements and increased germ cell death. Extended delay before fixation and freezing affected many endpoints in the mink testicular tissue. Some of these endpoints may mimic chemically induced effects, which is important to consider when evaluating specimens from wild animals for environmental toxicity.

## Introduction

There is growing concern that male reproduction is affected by chemicals in the environment. Chemical cocktails are more likely to cause this damage than single compounds. One way to determine such changes is to use wild mink as a monitor species and evaluate testicular toxicity using the most sensitive method, histopathology [1,2]. Since prolonged time prior to fixation may be a problem when using wild animals [3], it is of great importance to evaluate how this affects the validity of histopathology of the testis. This was the main aim of the present study.

A number of studies have linked adverse male reproduction in humans [4,5], mammals, birds and fish [3,6–8] to environmental pollutants. A large number of these pollutants mimic the action of reproductive hormones [9,10] and are therefore referred to as endocrine-disrupting chemicals (EDCs). To detect adverse effects on male reproduction, histopathology of testicular tissue is considered the most sensitive tool [1]. During spermatogenesis, stem cells (spermatogonia) constantly divide in a dynamic organised process, resulting in motile haploid spermatozoa. It is known that various steps in spermatogenesis are affected by EDCs [11], but the complexity of testicular tissue makes histopathological evaluation difficult and time-consuming [1,2,12,13]. Image analysis has been discussed recently as a future necessary tool in quantitative evaluation of histopathology to overcome the subjectivity of manual evaluation and accelerate the process [14].

In laboratory studies, the effect of a single chemical on male reproduction is commonly investigated. However, during recent years there has been increasing interest in the effect of interactions between chemicals found in the environment, which has been named the “cocktail effect”. New chemicals are continually being produced and the effect of the resulting chemical cocktail is unclear and will vary depending on the environment. The recent report from the World Health Organisation on endocrine disruption underlined this issue and urged future investigations to take it into consideration when studying reproductive disturbances in humans and wildlife [15]. One way of confirming the effect is by collecting samples from wildlife and analysing them for signs of disturbed reproduction. This provides a good picture of how the complex mixture of chemicals can affect humans and wildlife. Using wild animals, in particular top predators, to study the effect of EDCs on male reproductive disorders has been suggested [15].

Mink has been suggested as a suitable sentinel species in environmental monitoring [16,17]. Mink is a semi-aquatic top predator that can accumulate certain chemicals and is sensitive to their toxic effects [16,18]. The animals can easily be housed in the laboratory if a controlled experimental setting is needed. Tissues for histopathological studies should ideally be placed in fixative directly post mortem. When working with wild animals this is almost impossible to achieve, as capture is usually both time-consuming and difficult, and transport to the laboratory may take several hours or days post mortem. For practical reasons the animals are sometimes even frozen [17]. This results in a variety of post-mortem changes before the animals can be investigated and the tissue placed in fixative. Problems with delayed fixation have been discussed in previous environmental studies [3,19]. It is highly important that methods to separate post mortem histological changes caused by delayed fixation from pre-mortem pathological changes are devised. The aim of this study was therefore to establish the effect of pre-fixation delay and freezing on mink testicular endpoints such as gross morphology (body weight, testicular weight and length) and histopathology (morphology and morphometric measurements), in order to determine robust endpoints in suboptimally fixed testicular tissue.

## Materials and Methods

### 2.1 Animals and gross morphology

Thirty healthy, 10-month-old, sexually mature mink (*Neovison vison*) from a commercial fur farm (Skyberga lantbruksprodukter AB, Kumla) in Sweden were collected at culling. One mink at a time was euthanised at the fur farm using carbon dioxide (95 vol.%) in a specially constructed euthanisation box until cardiac arrest, followed by pelt ablation. This euthanisation process conforms with EU legislation and Swedish Board of Agriculture standards [20]. The farmer euthanized all reproductive active male mink at the time of sample collection for the present study. He was blinded to the treatment groups and decided the order of animals to be euthanized. Post mortem, the mink were divided into six treatment groups, depending on time and treatment between euthanisation and fixation/ measurements. The first five mink euthanised were placed in treatment group 0 h, the next five in treatment group 6 h and so on.

Treatment group 0 h (n = 5): Directly after the pelt was removed, body weight, left testis weight and length were measured, one animal at a time, followed by fixation of left testis tissue (within 10 minutes post mortem).

Treatment groups 6, 18, 30, 42 h and 6 h + frozen (n = 5 per group): After euthanisation and pelt ablation, the animals were transported intact to the laboratory and left at room temperature (21°C) for 6, 18, 30 or 42 h. Measurements of body weight, left testis weight and length and fixation of left testis tissue were performed after 6, 18, 30 and 42 h. The five animals in the last group (6 h + frozen) were frozen (-20°C) 6 h post mortem and then thawed at room temperature (21°C) for 30 h prior to measurements of body weight, left testis weight and length and fixation of left testis tissue.

Absolute testicular weight was used instead of relative weight, because the testes are less influenced by body weight than most other tissues [2,21]. No ethical approval was required due to the use of offal from routinely culled mink from a commercial fur farm. No animals were killed for the purposes of this study only and the commercial fur farm approved the use of the mink for the study.

### 2.2 Histopathological sample preparation and staining

Testicular tissue is known to be difficult to fix and traditional formalin is discouraged due to its poor preservation of morphological details [1,2]. Bouin’s fluid has been a standard fixative for testicular tissue, but it has health and safety hazards [22]. Modified Davidson’s fluid, which consists of 30% 37–40% formaldehyde, 15% ethanol, 5% glacial acid and 50% distilled water, has been suggested as a superior substitute to Bouin’s [22] and is recommended by the Society of Toxicologic Pathology [2].

Two transverse tissue slices (approximately 2 mm thick) were cut in serial fashion from the left testis and fixed in modified Davidson’s fluid for 24 h at 4°C. They were then rinsed and stored in phosphate buffer until all 30 mink had been fixed. The testis slices were then trimmed to appropriate size with a razor blade to fit in the embedding cassettes, followed by dehydration in increasing concentrations of ethanol. Testis slices fixed in modified Davidson’s fluid were used for both resin and paraffin embedding.

For morphological evaluations, one testis slice from each mink, was embedded in a water-soluble resin (Leica Historesin, Heidelberg, Germany) and sectioned with glass knives into 2 μm thin sections using a microtome (Leica RM 2165, Leica Instruments, Germany). All sections were stained with haematoxylin, eosin (HE) and toluidine.

For immunohistochemistry and semi-automatic image analysis, one testis slice from each animal was embedded in paraffin wax and cut into 4 μm thin sections using a sledge microtome (Leitz Wetzlar, 1400, Germany). One testis section from each mink was mounted on Superfrost Plus Gold slides (Menzel-Glaser, Braunschweig, Germany) for immunohistochemistry and one section was placed on Superfrost slides (Menzel-Glaser, Braunschweig, Germany) for image analysis. For the image analysis, the sections were stained with a variant of periodic acid-Shiff staining to obtain maximum colour differences between the seminiferous epithelium and the interstitial tissue.

#### 2.2.1 Variant of periodic acid-Shiff stain

The sections were deparaffinised in xylene (twice for 5 minutes each) and rehydrated (100% (twice), 95% and 70% ethanol for 5 minutes each, followed by distilled water), incubated for 5 minutes at room temperature (21°C) in periodic acid (0.5%), rinsed in distilled water several times, incubated in Shiff’s reagent (40 minutes) and washed in running water for 25 minutes. To counterstain, the slides were submerged in Weigert for 10 seconds, followed by 7 seconds in a mix of 95% ethanol and 1% HCl, and then rinsed in running water for 30 minutes. Sections were dehydrated through rising ethanol concentrations (70%, 95%, twice at 100%, 5 minutes at each concentration), transferred into xylene (three times, 10 minutes in total) and mounted with Pertex.

#### 2.2.2 Immunohistochemistry

Immunohistochemical localisation of Gata-4 antibody was evaluated as a marker to distinguish between different cell types in the seminiferous epithelium of the testis. The slides were deparaffinised in xylene (three times, 5 minutes each) and rehydrated (100% (twice), 95% and 70% ethanol for 2 minutes at each concentration, followed by distilled water (twice) for 5 min each). Antigen were retrieved by submerging the slides in 0.01 M sodium citric buffer (pH 6.0), followed by pressure-heating for 20 minutes in a pressure boiler at 120°C (21100 Retriever, Histolab Products AB, Gothenburg, Sweden). After cooling, the slides were rinsed in phosphate-buffered saline (PBS). The PBS used for the entire immunoprotocol had pH 7.4 and contained 0.8% NaCl, 0.02% KCl, 0.106% Na_2_HPO_4_ and 0.02% KH_2_PO_4_. The endogenous peroxidase activity was blocked using 0.3% hydrogen peroxide diluted in methanol. Immunolocalisation of GATA-4 antibody was identified using the ImmunoCruz goat ABC Staining System (sc-2023, Santa Cruz Biotechnology, Santa Cruz, CA). In brief, tissue sections were treated with blocking serum goat (sc-2023, Santa Cruz Biotechnology, Santa Cruz, CA) for 30 minutes and excess serum was blotted from the slides. GATA-4 (C20) (sc-1237, Santa Cruz Biotechnology, Santa Cruz, CA) antibody was diluted in PBS 1:50 and the sections were incubated in the dark for 20 h at 4°C. The sections were rinsed with PBS between each of the subsequent steps. Secondary antibody (donkey anti-goat) (sc-2023, Santa Cruz Biotechnology, Santa Cruz, CA) was applied to each section and incubated for 30 minutes, followed by AB enzyme reagent for 30 minutes. Immunoreactivity was visualised using 3.3´-diaminobenzine tetrahydrochloride (DAB Safe, Saveen Biotech, Malmö, Sweden), to which H_2_O_2_ was added to visualise the bound enzyme activity as brown colour. Sections were finally rinsed in H_2_O, dehydrated and mounted with Pertex. Negative controls were run by excluding the primary antibody on one slide and by replacing the primary antibody with non-immune serum from goat (Goat Ig G, Sc-2028) on one slide. The slides were counterstained with haematoxylin for 10 seconds.

### 2.3 Morphological evaluation

#### 2.3.1 HE Sections

One person (E. Sporndly-Nees) performed the morphological evaluation of the cells in the seminiferous tubules. The evaluation was based on both visual comparison of unblinded slides under the microscope and comparison of digital images (taken with a Nikon Microphot-FXA microscope using 10x, 20x, 40x and 60x objective lenses). A total number of 649 images were taken (0 h: 73 images; 6 h: 106 images; 18 h: 132 images; 30 h: 137 images; 42 h: 115 images; and 6 h + frozen: 86 images). Factors compared between the treatment groups were overall organisation of seminiferous epithelium and tubular lumen at 10x and 20x magnification. To compare cellular details (size, shape and nuclear pattern), 40x and 60x magnifications were used. The specific cell types studied in the seminiferous epithelium were: Sertoli cells, spermatogonia, primary spermatocytes (zygotene, leptotene and pachytene), round spermatocytes, elongated spermatids and Leydig cells. One cell type at a time was studied within each group (0, 6, 18, 30 and 42 h and 6 h + frozen) and described. When all cell types within each group had been described, a comparison was made between the groups to describe how the cell morphology changed over time and by freezing. Staging of the seminiferous tubules were carried out, when possible, in all sections.

#### 2.3.2 Immunohistochemical sections

One person (E. Sporndly-Nees) performed the morphological evaluation of the immunohistochemical sections. The evaluation was based on visual comparison of unblinded slides under the microscope using 10x, 20x, 40x and 60x objective lenses. Gata-4 stained Sertoli cells and acrosomes were identified when possible.

### 2.4 Morphometric measurements

Digital images of paraffin sections were taken with a Nikon Microphot-FXA microscope using a 10x objective lens. A series of images from each animal were taken in a grid-like pattern to make sure that no tubular overlap occurred. A total of 1022 round-shaped, straight-cut seminiferous tubules were evaluated. Mean (range) number of seminiferous tubules measured in each group was: 0 h: 61.4(37–93); 6 h: 31.6(20–46); 18 h: 30.4(21–38); 30 h: 29.4(14–50); 42 h: 29.8(13–38); and 6 h + frozen: 21.8(13–26). Morphometric measurements of area, diameter and epithelial height of seminiferous tubules were analysed by semi-automatic image analysis using a method designed specifically for testicular tissue (below). The measurements were performed by one investigator (A. Fakhrzadeh) and all images were coded.

#### 2.4.1 Semi-automatic image analysis

A semi-automatic image analysis technique was developed to measure area, epithelial height and diameter of the seminiferous tubules. A semi-automated programme that includes user input to produce a correct delineation was used to find the boundaries of the seminiferous tubules. The programme determines various measures for each pixel belonging to an edge. The gradient image has information about edges and was computed using the vector gradient approach [23]. From the gradient image, the gradient magnitude and gradient direction were calculated. The Laplacian zero crossing [24] and Canny edge detector [25], both implemented in the Matlab edge detector function, were also used. These four measures were combined by a weighted average into a single cost function:

$$c\left(p,q\right)=4f_{z}\left(q\right)+4f_{c}\left(q\right)+f_{g}\left(q\right)+f_{D}\left(p,q\right)$$

Where $f_{z}\left(q\right)$, $f_{c}\left(q\right)$, $f_{G}\left(q\right)$ and $f_{D}\left(q\right)$ were the magnitudes obtained by the zero crossing, Canny edge detector, gradient magnitude and gradient direction, respectively.

This cost function was plugged into the livewire algorithm [26,27] implemented in Matlab [28]. The user added a seed point on the boundary of a tubule and the algorithm calculated the cost of the optimal boundary between this seed point and all other pixels in the image. By moving the mouse over the image, the user could then instantly see the optimal boundary between that seed point and the mouse position. By clicking, that portion of the boundary became fixed, and the newly selected pixel became the seed point, repeating the whole process. In this manner, the user could, interactively and with only a few clicks, very precisely delineate the whole tubule boundary.

After delineating a tubule, its area was given by the number of the pixels inside the drawn boundary, and its radius was computed as the average distance from all pixels on the drawn boundary to the tubule’s centre of mass. For the tubules with visible lumen, the lumen was segmented using the same procedure as described above. The epithelium height was then computed as the average distance between the border of the lumen and the border of the tubule.

### 2.6 Statistical analysis

The experimental unit was mink. The gross morphology (body weight (kg), left testis weight (g) and length (mm) was based on one measurement per mink and therefore analysed by one-way ANOVA. The morphometric measurements (area (mm^2^), diameter (μm) and epithelial height (μm) of seminiferous tubules) were based on several measurements per mink and a hierarchical ANOVA was therefore used. Two models were used, one including the fixed effect of time (0, 6, 18, 30 and 42 h) and the other the effect of freezing (0 and 6 h, 6 h + frozen). The analyses were carried out with SAS software (SAS Institute Inc., Cary, NC, USA, version 9.3) using the MIXED procedure. Residuals were analysed and were considered normally distributed with equal variances. Significance levels for pair-wise comparisons were corrected for multiple testing using Tukey’s method. All values presented in diagrams represent mean±S.E. and results were considered significant at p<0.05.

## Results

### 3.1 Gross morphology

Body weight and testicular length were not affected by prolonged storage time before fixation (6, 18, 30 or 42 h) or by freezing prior to measurements (6 h + frozen group) (Tables 1 and 2). Testicular weight was reduced by freezing compared with the samples measured immediately (0 h) (p = 0.049) and 6 h post mortem (p = 0.029). Testicular weight was not affected by delayed fixation (Table 2).

**Table 1:** Effect of freezing post mortem on measurements of body weight, testicular length, testicular weight, tubular area, tubular diameter and epithelial height in mink.

	0 h (n = 5)	6 h (n = 5)	6 h + frozen (n = 5)	Num DF	Den DF	F- value	P-value
**Body weight (kg)**	1.34±0.06^a^	1.41±0.06^a^	1.40±0.06^a^	2	12	0.37	0.697
**Testicular length (cm)**	2.11±0.09^a^	2.27±0.09^a^	2.05±0.09^a^	2	12	1.79	0.209
**Testicular weight (g)**	3.28±0.19^a^	3.36±0.19^a^	2.57±0.19^b^	2	12	5.39	0.021
**Tubular area (mm** ^**2**^ **)**	0.237±0.011^a^	0.251±0.011^a^	0.186±0.012^b^	2	12.4*	8.44	0.005
**Tubular diameter (μm)**	549.8±13.3^a^	565.0±14.0^a^	486.9±14.7^b^	2	12.5*	8.26	0.005
**Epithelial height (μm)**	169.0±4.13^a^	282.5±6.7^b^	243.5±7.1^c^	1	8.04*	16.10	0.004

One-way ANOVA was used to analyse body weight, testicular length and weight. Hierarchical ANOVA was used to analyse epithelial height, tubular area and diameter.

*Decimals due to Kenward-Roger adjustment.

Within each row, values with the same letter in superscript (^a^, ^b^ or ^c^) do not differ significantly (*P*>0.05). Values expressed as mean ± standard error of mean. Num = Numernator. Den = Denominator. DF = Degree of freedom.

**Table 2:** Effect of different time intervals post mortem on measurements of body weight, testicular length, testicular weight, tubular area, tubular diameter and epithelial height in mink.

	0 h (n = 5)	6 h (n = 5)	18 h (n = 5)	30 h (n = 5)	42 h (n = 5)	Num DF	Den DF	F- value	P-value
**Body weight (kg)**	1.34±0.06^a^	1.41±0.06^a^	1.48±0.06^a^	1.51±0.06^a^	1.58±0.06^a^	4	20	2.53	0.073
**Testicular length (cm)**	2.11±0.09^a^	2.27±0.09^a^	2.07±0.09^a^	2.18±0.09^a^	2.12±0.09^a^	4	20	0.69	0.609
**Testicular weight (g)**	3.28±0.21^a^	3.36±0.21^a^	2.92±0.21^a^	3.00±0.21^a^	2.82±0.21^a^	4	20	1.25	0.321
**Tubular area (mm** ^**2**^ **)**	0.237±0.013^ab^	0.251±0.020^a^	0.206±0.014^ab^	0.246±0.014^ab^	0.192±0.014^b^	4	19.9*	3.53	0.025
**Tubular diameter (μm)**	550.0±17.5^a^	564.8±18.0^a^	513.5±18.0^a^	560.0±18.2^a^	489.8±18.1^a^	4	20	3.26	0.033
**Epithelial height (μm)**	161.8±11.6^a^	282.4±11.9^b^	256.8±11.9^b^	280.0±12.0^b^	245±11.9^b^	4	20.4*	17.70	<0.001

One-way ANOVA was used to analyse body weight, testicular length and weight. Hierarchical ANOVA was used to analyse epithelial height, tubular area and diameter.

*Decimals due to Kenward-Roger adjustment.

Within each row, values with the same letter in superscript (^a^, ^b^ or ^ab^) do not differ significantly (*P*>0.05). Values expressed as mean ± standard error of mean. Num = Numernator. Den = Denominator. DF = Degree of freedom.

### 3.2 Histopathology

#### 3.2.1 Morphometric measurements

The area of seminiferous tubules decreased over time and after freezing (Tables 1 and 2). Tubular area was constant for up to 30 h of delayed fixation, but at 42 h the area decreased compared with the tissue fixed 6 h post mortem (p = 0.048). Freezing the tissue decreased the tubular area compared with the tissue fixed immediately (0 h) (p = 0.020) and the tissue fixed 6 h post mortem (p = 0.006). Tubular diameter also decreased when the testicular tissue had been frozen prior to fixation, compared with that in the tissue fixed immediately post mortem (0 h) (p = 0.020) and the tissue fixed 6 h post mortem (p = 0.006). Tubular diameter decreased over time (Table 2) and showed a tendency to decrease (p = 0.055) between the tissue fixed 6 h and 42 h post mortem. Epithelial height was affected by time (Table 2) and showed a significant increase in all groups (6, 18, 30 and 42 h, 6 h + frozen) compared with 0 h. To distinguish between the effects of time and freezing on epithelial height, a comparison of the 6 h and 6 h + frozen groups was performed, since the epithelial height increased significantly already after 6 h of delayed fixation. A hierarchical ANOVA was used. This comparison revealed significantly lower epithelial height in the 6 h + frozen group compared with the non-frozen 6 h group (Table 1).

#### 3.2.2 Morphology in HE sections

**Fig 1:**
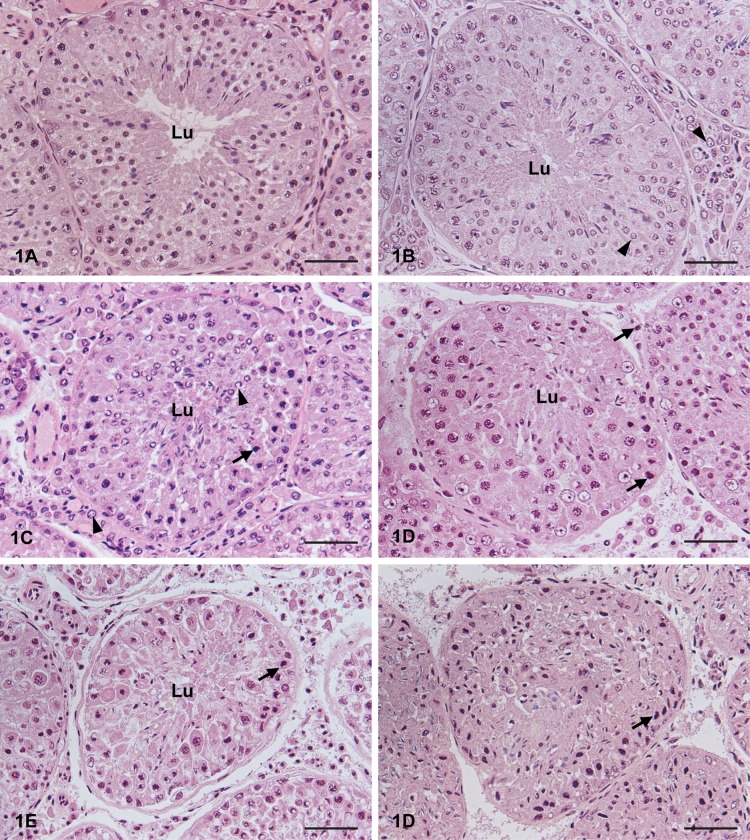
Time and freezing effects on morphological organisation in mink seminiferous tubules. Cross-sections of mink testis tissue with 0, 6, 18, 30 and 42 h delay before fixation, or frozen after 6 h and thawed prior to fixation. (a) Mink testis fixed immediately (0 h) post mortem. An organised tissue with an intact epithelium and a well-defined lumen in the seminiferous tubules. Germ cells are well connected and attached to the basement membrane. (b) Mink testis with a 6 h delay before fixation. The epithelium is well-organised and germ cells attached to the basement membrane. Lumen (Lu) of the seminiferous tubule is filled up with excess cytoplasm. Chromatin changes in round spermatids and Leydig cells can be seen (arrowheads). (c) Mink testis with an 18 h delay before fixation. The seminiferous tubular epithelium shows increased disorganisation, but the peritubular cells are attached to the seminiferous epithelium. Lumen (Lu) of the seminiferous tubule is occupied by cytoplasm, mixed with round and elongated spermatids. Chromatin changes such as pyknosis (arrow: spermatocyte) and chromatin margination and nuclear clearing (arrowhead: round spermatid and Leydig cell) can be seen. (d) Mink testis with a 30 h delay before fixation. The seminiferous epithelium is further disorganised, with germ cells located in abnormal positions. The peritubular cells are mostly attached to the seminiferous epithelium, but detached cells are visible. Chromatin changes such as pyknosis (arrow) can be seen, which makes identification of some cells difficult. (e) Mink testis with a 42 h delay before fixation. It is difficult to distinguish between the different cell types in the seminiferous epithelium due to pyknotic nuclei (arrow). There is a loss of intercellular connections, which contributes to distance and disorganisation between the germ cells. The peritubular cells are detached from the seminiferous epithelium. (f) Mink testis frozen after 6 h and thawed prior to fixation. A complete loss of organisation in the seminiferous tubular epithelium can be seen. The seminiferous epithelium resembles a dense mass. All cell nuclei show condensed chromatin and appear pyknotic (arrow). All testis samples were fixed in modified Davidson's fluid and stained with HE. Bar = 50 μm.

**Fig 2:**
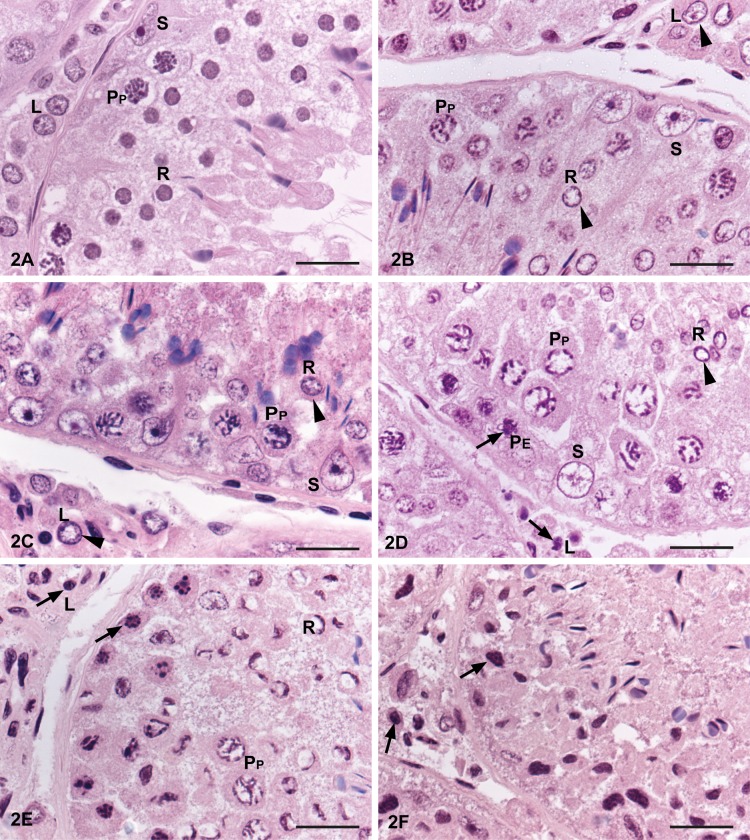
Time and freezing effects on detailed cell morphology in mink seminiferous tubules. Morphological alteration of nuclei in mink testis tissue samples with 0, 6, 18, 30, 42 h delay prior to fixation or frozen 6 h post mortem and thawed prior to fixation. (a) Mink testis fixed 0 h post mortem show normal morphology and different germ cells are easy to distinguish. Sertoli cells (S) show angular nuclei with heterochromatin and a dense nucleolus, with the nucleus in close proximity to the basement membrane. The interstitial tissue shows Leydig cells (L) and capillary and lymphatic vessels are easy to recognise. (b) Mink testis with a 6 h delay before fixation. Round spermatids (R) and Leydig cells (L) (arrowhead) express chromatin margination and nuclear clearing. Sertoli cells (S) show a lighter, rounder, slightly enlarged nucleus with partially condensed chromatin pattern. Pachytene spermatocytes (PP) with chromatin clumping and slightly enlarged nuclei. (c) Mink testis with an 18 h delay before fixation. Round spermatids (R) and Leydig cells (L) show nuclei clearing and chromatin margination (arrowhead). Sertoli cells (S) with rounder, further enlarged nuclei with fragmented chromatin pattern. (d) Mink testis with a 30 h delay before fixation. Primary spermatocytes (P) show chromatin rearrangement to a greater extent with chromatin condensation in early primary spermatocytes (PE) (arrow) or chromatin clumping and clearing of the cytoplasm in pachytene spermatocytes (PP). The chromatin of the round spermatids (R) shows a similar pattern as at 18 h, with nuclear clearing and chromatin margination (arrowhead), but most of the Leydig cells (L) show pyknotic nuclei (arrow). Sertoli cells are frequently separated from the basement membrane, with enlarged and vacuolated nuclei. (e) Mink testis with a 42 h delay before fixation. It is difficult to distinguish between the different cell types. A few pachytene primary spermatocytes (PP) are identifiable with lighter nucleus. Early primary spermatocytes are difficult to distinguish from spermatogonia, as their nuclei are pyknotic (arrow). The chromatin of the round spermatids (R) shows chromatin rearrangement in a dense, half-moon shape. In the interstitium, all cells have a pyknotic appearance (arrow) and it is not possible to identify different cell types. (f) Mink testis frozen after 6 h and thawed prior to fixation. It is not possible to identify different cell types, while all cell nuclei show condensed chromatin with pyknotic appearance (arrow). The exception is the elongated spermatids that can be identified. Bar = 20 μm. S = Sertoli cell, R = round spermatid, L = Leydig cell, P_E_ = Early primary spermatocyte (leptotene and zygotene), P_P_ = Pachytene spermatocyte. All testis samples were fixed in modified Davidson's fluid and stained with HE.

At 0 h post mortem, the tissue was well-organised, with an intact epithelium and a well-defined lumen in the seminiferous tubules. Germ cells were well connected and attached to the basement membrane (Fig 1a). Germ cells presented good nuclear morphology and different cell types were easy to identify. Sertoli cells had angular nuclei with heterochromatin and a dense nucleolus, with the nucleus in close proximity to the basement membrane. The interstitial tissue was well-organised and Leydig cells and capillary and lymphatic vessels were easy to identify (Fig 2a). Staging of the seminiferous epithelium was performed according to Pelletier [29].

At 6 h post mortem, many of the seminiferous tubular lumen were occupied, mostly by excess cytoplasm but also by round and elongated spermatids, and only a few empty lumen were seen. The epithelium was well-organised and germ cells were attached to the basement membrane (Fig 1b). Overall, spermatogonia had similar morphology as at 0 h, but a few cells showed a partly condensed chromatin nuclear pattern. Primary spermatocytes generally had normal morphology, but initial signs of autolysis had appeared, with chromatin condensation and pyknosis (early primary spermatocytes: zygotene and leptotene) or enlarged nuclei with chromatin clumping (pachytene primary spermatocytes). Round spermatids and Leydig cells showed similar morphology as at 0 h, but some cells had clearing in nuclei and chromatin margination. Elongated spermatids were well preserved. Sertoli cell presented a lighter nucleus and partially condensed chromatin pattern with a rounder, slightly enlarged shape (Fig 2b). Staging of the seminiferous epithelium was possible.

At 18 h post mortem, the seminiferous tubular epithelium showed increased disorganisation, but the peritubular cells were still attached to the seminiferous epithelium. All lumen of the seminiferous tubules was occupied by cytoplasm as a blurred haze, sometimes mixed with round and elongated spermatids (Fig 1c). Spermatogonia showed overall normal morphology, but some cells were pyknotic. Primary spermatocytes and elongated spermatids showed similar patterns as at 6 h. Most round spermatids showed nucleus clearing and chromatin margination. Leydig cells showed the same pattern as at 6 h, but appeared slightly enlarged. The Sertoli cells resembled the 6 h samples but appeared further enlarged, with fragmented chromatin pattern and a tendency for rounder nuclei (Fig 2c). Staging of the seminiferous epithelium was possible, but stage 1–4 and stage 5–7 were difficult to separate.

At 30 h post mortem, the seminiferous epithelium was further disorganised, with germ cells located at abnormal positions (Fig 1d). The peritubular cells were mostly attached to the seminiferous epithelium, but detached cells were seen. Most spermatogonia were difficult to identify and those seen had condensed chromatin. Primary spermatocytes were mostly identifiable, but the chromatin had rearranged to a greater extent, with either chromatin condensation (early primary spermatocytes) or chromatin clumping and lightening of the cytoplasm (pachytene primary spermatocytes). The chromatin of the round spermatid showed a similar pattern as at 18 h, with nuclear clearing and chromatin margination. Elongated spermatids had normal morphology. In the interstitial tissue, Leydig cell nuclei were mostly pyknotic. Sertoli cells were frequently separated from the basement membrane, with enlarged and vacuolated nuclei, and occasionally the nucleolus was dissolved (Fig 2d). In some seminiferous tubules it was not possible to identify any Sertoli cells. Staging was difficult and only possible in larger stage categories.

At 42 h post mortem, the longest interval studied, it was difficult to distinguish between the different cell types in the seminiferous epithelium. There was a loss of intercellular connections, which contributes to distance and disorganisation between the germ cells. The peritubular cells were detached from the seminiferous epithelium in many tubules (Fig 1e). Only a few spermatogonia with condensed chromatin pattern were identifiable. A few primary spermatocytes were recognisable and they had either a lighter nucleus with partly condensed chromatin or condensed pyknotic nuclei. The chromatin of the round spermatids was now rearranged into a dense, half-moon shape. Elongated spermatids were possible to distinguish, but the location was disorganised. In the interstitium, all cells had a pyknotic appearance and it was not possible to identify cell types, but vessels were identifiable (Fig 2e). Sertoli cells were only occasionally identifiable. Staging was not possible.

Testicular tissue frozen 6 h post mortem and thawed before fixation showed a complete loss of organisation in the seminiferous tubular epithelium. The seminiferous tubules were still identifiable, but resembled a dense mass in which peritubular cells were visible but detached from the epithelium. All cell nuclei had condensed chromatin and appeared pyknotic (Fig 1f). The exception was elongated spermatids, which could be identified on some occasions. In the interstitial tissue some vessels were identifiable, but the Leydig cells were not distinguishable as all cell nuclei had condensed chromatin (Fig 2f). Staging was not possible.

#### 3.2.3 Morphology in Gata-4 stained sections

**Fig 3:**
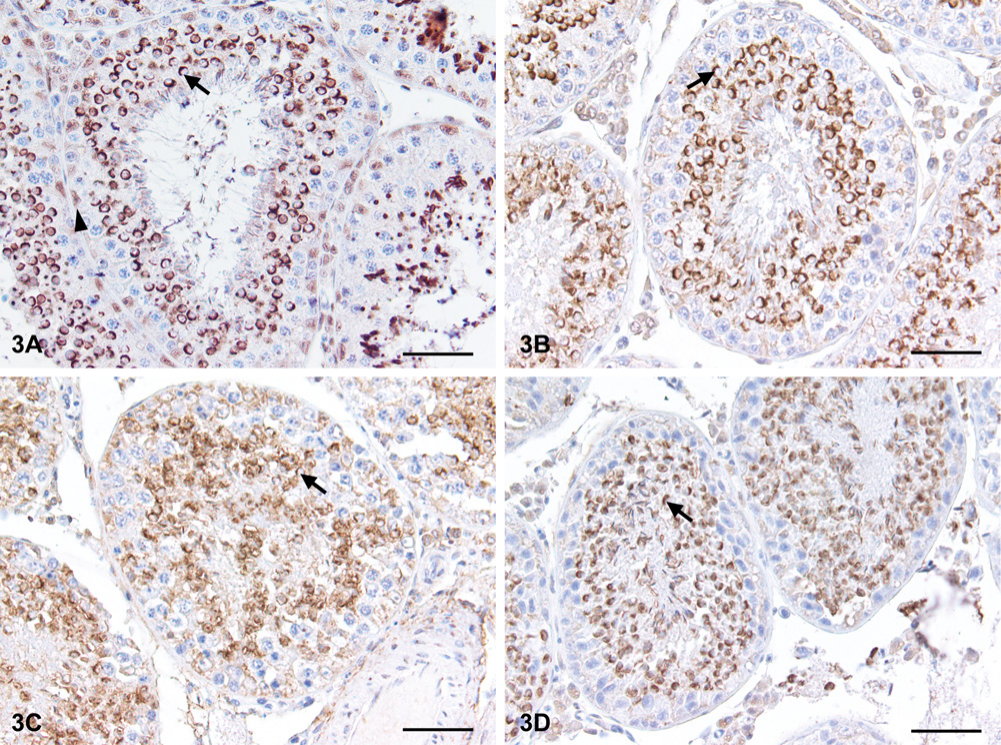
Time and freezing effects on immunohistochemical localisation of Gata-4 in mink testicular tissue. Immunohistochemical localisation of Gata-4 in mink seminiferous tubules with a delay of 0, 6 and 42 h post mortem before fixation, or frozen 6 h post mortem and thawed prior to fixation. (a) Mink testis fixed immediately post mortem. Sertoli cells (arrowhead) stain light brown for Gata-4. Acrosomes (arrow) are stained dark brown. (b) Mink testis fixed after 6 h of storage post mortem. Sertoli cells show no staining but acrosomes (arrow)s are stained dark brown. The seminiferous epithelium is well organised. (c) Mink testis fixed after 42 h of storage post mortem. Note the increased disorganisation of the seminiferous epithelium. The acrosomes are still stained dark brown (arrow), but background stain has increased. (d) Mink testis frozen after 6 h and thawed prior to fixation. The acrosomes are stained dark brown. Bar = 50 μm. All testes samples were fixed in modified Davidson's fluid and weak haematoxylin counterstain.

The tissue fixed directly post mortem (0 h) showed distinct staining for Gata-4 of Sertoli cell nuclei in the seminiferous epithelium (Fig 3a). In addition, a dark and distinct acrosome stain of round and elongated spermatids was seen, which enabled distinction between spermatids in Golgi, cap, acrosome and maturation phase. In the interstitial tissue, the nuclei of Leydig cells were stained with moderate intensity, while fibroblasts and endothelial cells remained unstained.

In all treatments (6, 18, 30, 42 h post mortem delay and 6 h + frozen), there was no staining of Sertoli cells. The acrosomes remained marked all through the different time periods and freezing (Fig 3b–3d). There was a change in distinctiveness of the acrosome stain over time, with increased difficulty in distinguishing which stained acrosome belonged to which nucleus. The nonspecific background stain and disorganisation of the seminiferous epithelium were amplified as the time between euthanisation and fixation increased.

## Discussion

### 4.1 Morphological changes may be misinterpreted as toxic damage if fixation is delayed

Testicular histopathology is the most sensitive and reliable method for detecting testicular toxicity [1,2]. In the present study we examined some endpoints frequently used in testicular histopathology to detect reproductive toxicity and how they are affected by prolonged storage before fixation. However, freezing the tissue prior to fixation altered the morphology so severely that no histopathological endpoints could be used and the tissue was not possible to evaluate. The morphology of frozen tissue is therefore not discussed further below.

One of the first signs of autolysis observed in histological examination of testicular tissue in the present study was shredded cytoplasm and germ cells occupying the seminiferous tubular lumen, which was apparent after only 6 h of storage between euthanisation and fixation. Shredding of epithelial content to the lumen of the seminiferous tubules can be a sign of tissue damage. A review by Creasy [30] noted that if any cell in the testicular tissue is affected, it generally results in exfoliation of the dead germ cell into the tubular lumen, regardless of the mechanism of toxicity. Lanning et al. [2] therefore recommend that a search for exfoliation of germ cells into the lumen of seminiferous tubules be performed in routine testicular histopathology. This is supported by several studies in which animals were exposed to toxicants such as cadmium, nickel and di (n-butyl) phthalate, reporting germ cells sloughing into the tubular lumen as a sign of toxic damage [31–33]. The results from the present study indicated that signs of exfoliated germ cells and cytoplasm in tubular lumen should be used with caution as endpoints if the delay prior to fixation is prolonged, while it may be misinterpreted as toxic damage. In a study examining time-dependent post mortem histopathology in rat testes, Bryant and Boekelheide [34] observed no sloughing of the apical seminiferous tubular epithelium, even after 48 h.

In a review paper, Mathur and D'Cruz [35] state that EDCs induce DNA fragmentation and chromatin damage, resulting in impaired testicular function. In the present study, chromatin rearrangement and pyknotic nuclei were frequently observed. Round spermatids and Leydig cells showed initially nuclear lightening and chromatin margination. At 30 h post mortem, the chromatin of round spermatid rearranged in a dense, collapsed half-moon shape. Leydig cell nuclei appeared enlarged at 18 h, followed by condensed chromatin and pyknosis at 30 h post mortem. Some spermatogonia and primary spermatocytes were pyknotic already at 6 h. Toxicologically induced dying spermatocytes show nuclear pyknosis, while round spermatids express chromatin margination [2]. These descriptions are consistent with what we found in the present study, and could be misinterpreted as toxic damage if fixation has been delayed. Furthermore, according to Lanning et al. [2], the most sensitive marker for decreased testosterone levels in the testis is an increased number of degenerated pachytene spermatocytes and round spermatids at stage VII. An increase in pyknotic spermatocytes was observed in the present study already after 6 h of storage prior to fixation, and this endpoint must therefore be used carefully if pre-fixation time is delayed. Rats show a higher incidence of apoptotic (TUNEL-positive) spermatogenic cells after exposure to a moderate level of di (n-butyl) phthalate A and complete germ cell loss at a higher dose [31]. In a study of time-dependent morphological changes in rats, pyknosis was seen in spermatogonia type A after 36 h and in Leydig cells after 24 h post mortem [34]. Those authors commented that Leydig cell death is rather unusual for most toxicological studies. However, the morphological appearance of the Leydig cell is not considered a sensitive indicator of its function [2]. In the present study pyknosis of Leydig cells was also observed after 30 h, which corresponds well with Bryant and Boekelheide [34], but spermatogonia pyknosis appeared as early as 6 h post mortem.

Toxic damage can lead to germ cell death, but the dead germ cells are phagocytised by Sertoli cell within 24 h, resulting in generalised germ cell depletion [2]. Total germ cell depletion was not seen in the present study. However, chromatin rearrangement in germ cells was observed already after 6 h of storage and this can make it difficult to distinguish and count cells. The reduction in germ cells per testis or in Sertoli cells and Sertoli cell only tubules was used as the endpoint in a previous study evaluating how sewage sludge chemicals affected sheep exposed in utero and post natum and the results supported the use of this endpoint [7]. No specific germ cell loss or stage-related effects are expected in chronic exposure to a testicular toxicant owing to the duration and effect of maturation [2]. Wild animals can have been exposed in both the long and short term to various EDCs, which complicates the evaluation.

It is recommended that numbers of vacuoles within the tubular epithelium be examined [2]. No vacuolisation of the seminiferous tubular epithelium was seen in the present study with up to 42 h of storage before fixation. This confirms findings by Bryant and Boekelheide [34] in a study of prolonged storage of rat testes, which further supports use of the endpoint. Sitka black-tailed deer in Alaska display focal vacuolisation in the testicular tissue, an effect that could be attributable to environmental endocrine disruption [36].

### 4.2 Gross morphology was not altered by 42 h pre-fixation delay

This study showed that testicular weight was not affected by storage of mink carcasses for up to 42 h, but that it was significantly reduced by freezing the animals. Testicular weight is a known measure of reproductive health, with a decrease generally indicating germ cell loss or impaired spermatogenesis associated with decreased fluid production by the Sertoli cell [1,2]. Testicular weight is therefore a frequently used endpoint in controlled laboratory studies of the impact of EDCs on reproduction. According to Ryokkynen et al. [37], oestrogenic substances such as phytoestrogens increase testicular weight in male mink kits exposed during gestation and lactation. In male rats exposed to environmental oestrogens in utero and during neonatal development, testicular weight decreases as they reach adult age [38]. The results obtained in the present study support the use of testicular weight as a parameter even if the animal has been stored for up to 42 h between euthanisation and weighing of the testes. However, data from animals frozen before weight recording should be used with caution, as freezing affected the results in our study. Bryant and Boekelheide [34] investigated the influence of prolonged storage of testicular tissue in rats and reported a decrease in testicular weight over time, but did not evaluate the difference statistically.

Testicular length and body weight are both commonly used endpoints in toxicological studies. In the present study these endpoints remained constant even though the tissue had been exposed to prolonged storage for up to 42 h prior to measurements or to freezing, which confirms their applicability as endpoints. According to Sonne et al. [3], there is an inverse relationship between testis length in polar bears in East Greenland and organohalogen pollutants in their environment, which supports the use of this endpoint.

### 4.3 Diameter and area are more reliable endpoints than epithelial height

Epithelial height and diameter of the seminiferous tubules are frequently used parameters to evaluate male reproduction, as known environmental chemicals, such as cadmium, nickel and di (n-butyl) phthalate, affect these parameters [31–33]. In the present study we found that the area and diameter of the seminiferous tubules were constant up to 30 h of storage, but at 42 h they decreased compared with values measured in tissue fixed within 6 h post mortem. The epithelial height changed significantly between the tissue fixed immediately (0 h) and all other groups. The diameter of the seminiferous tubules did not change, but epithelial height increased significantly and all observed seminiferous lumen disappeared when fixation was delayed by 6 h or more. The most likely explanation for the disappearance of lumen is loss of cell-to-cell contact, resulting in the lumen being occupied by germ cells. Another explanation could be shrunken tubules, but that would result in decreased tubular diameter. The findings in the present study suggest that seminiferous tubule diameter and area are predictable measures up to 30 h of tissue storage, but that epithelial height should not be used when fixation has been delayed. None of these parameters is suitable for use if the animals have been frozen prior to fixation. Tissues samples should be examined for signs of dilation or contraction of the seminiferous tubules when evaluating testicular toxicity, as this is considered to be one of the most sensitive parameters of testicular damage [2,30]. Sonne et al. [3] used seminiferous tubular diameter as an endpoint to investigate the relationship between testicular damage and organohalogen pollutants in East Greenland polar bears, but had to exclude animals due to suboptimal fixation. However, the results from the present study suggest that the use of these endpoints may be valid after certain types of suboptimal fixation.

A recent editorial published in Nature Methods [14] notes that microscopy is undergoing a crucial evolution into a more quantitative and powerful technique with the help of bioinformatics, including image analysis. Different approaches for assessing testicular histopathology are described in the literature. The Society of Toxicologic Pathology therefore recommends spermatogenic cycle evaluation, adding that quantitative procedures are inappropriate for screening studies but can be valuable in follow-up studies with a specific objective in mind [1]. Quantitative evaluation of a certain type of damaged germ cell in the seminiferous epithelium involves difficulties, because these cells are constantly dividing, resulting in renewal of the epithelium. There is also a relatively large variation between individuals in some endpoints, which results in a need for many animals per treatment group in order to detect a difference between treatments [39]. In the present study, epithelial height, tubular diameter and tubular area were quantified using image analysis. In contrast to many other quantitative endpoints, the variations in tubular diameter or volume between individuals and species are relatively small [40], which makes these more reliable endpoints. The main advantage of using image analysis in quantification is the objectivity and the greater speed of analysis.

### 4.4 Gata-4 is an unpredictable Sertoli cell marker

In the present study, the polyclonar antibody Gata-4 stained Sertoli cell nuclei in mink testicular tissue fixed immediately post mortem. Gata-4 also stains Sertoli cells in bulls, mice, rats, dogs and boars [41–45], making it a suitable marker for easy detection of these cells. However, when the time post mortem and prior to fixation was prolonged to 6 h or more, no Gata-4 marked Sertoli cells were observed in the present study, which makes the marker unsuitable under difficult field conditions. Scudamore et al. [46] reviewed the effects of post mortem delay on immunohistochemical labelling and highlighted the importance of both the post mortem delay before fixation and the antibody used, as different antibodies react differently. Surprisingly, Gata-4 also distinctively marked the acrosomes in both round and elongated spermatids in the present study. The stain of acrosomes seems to differ between species. Polyclonal Gata-4 stains the acrosomes of round spermatids in rats and has been suggested for use to facilitate staging of the seminiferous epithelium [45]. This possible use of Gata-4, especially in environmental research, seems promising, since the marking of acrosomes was constant with an interval of up to 42 h between euthanisation and fixation in the present study. Staging was not possible up to 42 h because the nuclear morphology was too poor, but the Gata-4 stain extended the possibility of staging.

### 4.5 Species differences and study limitation

Before generalising the present findings to other animal species, the size of the animal, type of fur/ feathers and where the testes are situated should be considered, as these may affect autolysis. As an example, autolysis of human liver is slower than autolysis of rat liver at 18°C [47]. The appearance of autolysis can also change depending on the organ investigated [48], an effect which should be taken into consideration.

In the present study the group 6 h + frozen was chosen to show how freezing effected endpoints. Why we chose to leave the animals 6 h post mortem and prior freezing was to mimic the situation in the field where it can take the hunter several hours to get back with the animals before it can be frozen. Prolonged time prior to fixation affected epithelial height severely between 0 h and 6 h resulting in difficulties to separate the freeze and time effect, and a 0 h + frozen would have been desirable. To adjust for this limitation the 6 h + frozen was compared with 6 h and thereby the freeze effect was isolated.

## Conclusions

Knowledge of the interval between death of the animal and fixation of testicular tissue is important when interpreting the results of morphometric and histopathological analyses. Even when fixation is delayed for up to 30 h post mortem, certain endpoints are not affected, such as area and diameter of the seminiferous tubules, length and weight of the testis and acrosomes marked with Gata-4. Endpoints that proved to be affected already with a 6 h delay in post mortem storage were epithelial height, Sertoli cells marked with Gata-4 and morphology of the cells. In addition, some morphological changes due to delayed fixation may be misinterpreted as toxic damage. Freezing tissues prior to fixation severely affected most endpoints evaluated.

## Perspectives

Due to the finding that the interval between death of the animal and fixation may be crucial for obtaining valid results, future research should investigate other testicular endpoints and methods to validate their use if the pre-fixation time is delayed.

## References

[1] Creasy DM. Evaluation of testicular toxicology: a synopsis and discussion of the recommendations proposed by the Society of Toxicologic Pathology. Birth Defects Res B Dev Reprod Toxicol. 2003;68: 408–415. 14745990 10.1002/bdrb.10041

[2] Lanning LL, Creasy DM, Chapin RE, Mann PC, Barlow NJ, Regan KS, et al Recommended approaches for the evaluation of testicular and epididymal toxicity. Toxicol Pathol. 2002;30: 507–520. 12187942 10.1080/01926230290105695

[3] Sonne C, Leifsson PS, Dietz R, Born EW, Letcher RJ, Hyldstrup L, et al Xenoendocrine pollutants may reduce size of sexual organs in East Greenland polar bears (Ursus maritimus). Environ Sci Technol. 2006;40: 5668–5674. 17007124 10.1021/es060836n

[4] Dallinga JW, Moonen EJ, Dumoulin JC, Evers JL, Geraedts JP, Kleinjans JC. Decreased human semen quality and organochlorine compounds in blood. Hum Reprod. 2002;17: 1973–1979. 12151423 10.1093/humrep/17.8.1973

[5] Petrelli G, Figa-Talamanca I. Reduction in fertility in male greenhouse workers exposed to pesticides. Eur J Epidemiol. 2001;17: 675–677. 12086082 10.1023/a:1015511625099

[6] Fox G. Wildlife as sentinels of human health effects in the Great Lakes-St. Lawrence basin. Environ Health Perspect. 2001;109: 853–861. 11744503 10.1289/ehp.01109s6853 PMC1240620

[7] Bellingham M, McKinnell C, Fowler PA, Amezaga MR, Zhang Z, Rhind SM, et al Foetal and post-natal exposure of sheep to sewage sludge chemicals disrupts sperm production in adulthood in a subset of animals. Int J Androl. 2012;35: 317–329. 10.1111/j.1365-2605.2011.01234.x 22150464 PMC3440584

[8] Vos JG, Dybing E, Greim HA, Ladefoged O, Lambre C, Tarazona JV, et al Health effects of endocrine-disrupting chemicals on wildlife, with special reference to the European situation. Crit Rev Toxicol. 2000;30: 71–133. 10680769 10.1080/10408440091159176

[9] Sohoni P, Sumpter JP. Several environmental oestrogens are also anti-androgens. J Endocrinol. 1998;158: 327–339. 9846162 10.1677/joe.0.1580327

[10] Kelce WR, Stone CR, Laws SC, Gray LE, Kemppainen JA, Wilson EM. Persistent DDT metabolite p,p'-DDE is a potent androgen receptor antagonist. Nature. 1995;375: 581–585. 7791873 10.1038/375581a0

[11] Saradha B, Mathur PP. Effect of environmental contaminants on male reproduction. Environ Toxicol Pharmacol. 2006;21: 34–41. 10.1016/j.etap.2005.06.004 21783636

[12] Creasy DM. Pathogenesis of male reproductive toxicity. Toxicol Pathol. 2001;29: 64–76. 11215686 10.1080/019262301301418865

[13] Berndtson WE. The importance and validity of technical assumptions required for quantifying sperm production rates: a review. J Androl. 2011;32: 2–14. 10.2164/jandrol.109.008870 20671145

[14] Editorial. The quest for quantitative microscopy. Nat Methods. 2012;9: 627 22930824 10.1038/nmeth.2102

[15] Bergman A, Heindel JJ, Jobling S, Kidd KA, Zoeller RT. State of the science of endocrine disrupting chemicals 2012: an assessment of the state of the science of endocrine disruptors prepared by a group of experts for the United Nations Environment Programme and World Health Organization; Bergman A, Heindel JJ, Jobling SKKA, Zoeller RT, editors. Geneva, Switzerland: World Health Organization; 2013 xxv + 260 p.

[16] Basu N, Scheuhammer AM, Bursian SJ, Elliott J, Rouvinen-Watt K, Chan HM. Mink as a sentinel species in environmental health. Environ Res. 2007;103: 130–144. 16716289 10.1016/j.envres.2006.04.005

[17] Persson S, Brunstrom B, Backlin B-M, Kindahl H, Magnusson U. Wild mink (Neovison vison) as sentinels in environmental monitoring. Acta Vet Scand. 2012;54: S9.

[18] Kihlström JE, Olsson M, Jensen S, Johansson Å, Ahlbom J, Bergman Å. Effects of PCB and Different Fractions of PCB on the Reproduction of the Mink (Mustela vison). Ambio. 1992;21: 563–569.

[19] Bubenik GA, Jacobson JP. Testicular histology of cryptorchid black-tailed deer (Odocoileus hemionus sitkensis) of Kodiak island, Alaska. Z Jagdwiss. 2002;48: 234–243.

[20] Concil Regulation (EC) of 24 september 2009 on the protection of animals at the time of killing. 1099. Official Journal of the European Union The Council of the European Union. 2009.

[21] Bailey SA, Zidell RH, Perry RW. Relationships between organ weight and body/brain weight in the rat: what is the best analytical endpoint? Toxicol Pathol. 2004;32: 448–466. 15204968 10.1080/01926230490465874

[22] Latendresse JR, Warbrittion AR, Jonassen H, Creasy DM. Fixation of testes and eyes using a modified Davidson's fluid: comparison with Bouin's fluid and conventional Davidson's fluid. Toxicol Pathol. 2002;30: 524–533. 12187944 10.1080/01926230290105721

[23] Lee HC, Cok DR. Detecting boundaries in a vector field. IEEE Trans Signal Process. 1991;39: 1181–1194.

[24] Hildreth E, Marr D. Theory of Edge Detection. Proc R Soc Lond. 1980;207: 182–217. 10.1098/rspb.1980.0020 6102765

[25] Canny J. A Computational Approach to Edge Detection. IEEE Trans Pattern Anal Mach Intell. 1986;PAMI-8: 679–698. 21869365

[26] Barrett W, Mortensen E. Interactive live-wire boundary extraction. Med Image Anal. 1997;1: 331–341. 9873914 10.1016/s1361-8415(97)85005-0

[27] Falcão AX, Udupa JK, Samarasekera S, Sharma S, Hirsch BE, de Alencar Lotufo R. User-steered image segmentation paradigms: Live wire and live lane. Graph Models Image Process. 1998;60: 233–260.

[28] Chodorowski A, Mattsson U, Langille M, Hamarneh G. Color Lesion Boundary Detection Using Live Wire. Med Image: Image Process. 2005;5747: 1589–1596.

[29] Pelletier RM. Cyclic formation and decay of the blood-testis barrier in the mink (Mustela vison), a seasonal breeder. Am J Anat. 1986;175: 91–117. 3953473 10.1002/aja.1001750109

[30] Creasy DM. Review Article: Evaluation of Testicular Toxicity in Safety Evaluation Studies: The Appropriate Use of Spermatogenic Staging. Toxicol Pathol. 1997;25: 119–131. 9125770 10.1177/019262339702500201

[31] Alam MS, Ohsako S, Matsuwaki T, Zhu XB, Tsunekawa N, Kanai Y, et al Induction of spermatogenic cell apoptosis in prepubertal rat testes irrespective of testicular steroidogenesis: a possible estrogenic effect of di(n-butyl) phthalate. Reproduction. 2010;139: 427–437. 10.1530/REP-09-0226 19903717

[32] de Souza Predes F, Diamante MA, Dolder H. Testis response to low doses of cadmium in Wistar rats. Int J Exp Pathol. 2010;91: 125–131. 10.1111/j.1365-2613.2009.00692.x 20015210 PMC2965898

[33] Toman R, Massanyi P, Adamkovicova M, Lukac N, Cabaj M, Martiniakova M. Quantitative histological analysis of the mouse testis after the long-term administration of nickel in feed. J Environ Sci Health A Tox Hazard Subst Environ Eng. 2012;47: 1272–1279. 10.1080/10934529.2012.672130 22540651

[34] Bryant BH, Boekelheide K. Time-dependent changes in post-mortem testis histopathology in the rat. Toxicol Pathol. 2007;35: 665–671. 17676525 10.1080/01926230701459994

[35] Mathur PP, D'Cruz SC. The effect of environmental contaminants on testicular function. Asian J Androl. 2011;13: 585–591. 10.1038/aja.2011.40 21706039 PMC3739630

[36] Veeramachaneni DN, Amann RP, Jacobson JP. Testis and antler dysgenesis in sitka black-tailed deer on Kodiak Island, Alaska: Sequela of environmental endocrine disruption? Environ Health Perspect. 2006;114 Suppl 1: 51–59. 16818246 10.1289/ehp.8052 PMC1874179

[37] Ryokkynen A, Nieminen P, Mustonen AM, Pyykonen T, Asikainen J, Hanninen S, et al Phytoestrogens alter the reproductive organ development in the mink (Mustela vison). Toxicol Appl Pharmacol. 2005;202: 132–139. 15629188 10.1016/j.taap.2004.06.012

[38] Sharpe RM, Fisher JS, Millar MM, Jobling S, Sumpter JP. Gestational and lactational exposure of rats to xenoestrogens results in reduced testicular size and sperm production. Environ Health Perspect. 1995;103: 1136–1143. 8747020 10.1289/ehp.951031136 PMC1519239

[39] Berndtson WE. Replication needed to distinguish alterations in cell ratios, the frequency of individual stages of the cycle of the seminiferous epithelium, or the appearance of abnormalities in the testes of rodents, rabbits, or humans. J Androl. 2010;31: 593–606. 10.2164/jandrol.109.008920 20133966

[40] Berndtson WE. Replication needed to detect alterations in the composition of rodent, rabbit, or human testes via volume density approaches. J Androl. 2010;31: 607–616. 10.2164/jandrol.110.010116 20671142

[41] McCoard SA, Lunstra DD, Wise TH, Ford JJ. Specific Staining of Sertoli Cell Nuclei and Evaluation of Sertoli Cell Number and Proliferative Activity in Meishan and White Composite Boars During the Neonatal Period. Biol Reprod. 2001;64: 689–695. 11159374 10.1095/biolreprod64.2.689

[42] Ramos-Vara JA, Miller MA. Immunohistochemical evaluation of GATA-4 in canine testicular tumors. Vet Pathol. 2009;46: 893–896. 10.1354/vp.08-VP-0287-R-BC 19429994

[43] Ketola I, Anttonen M, Vaskivuo T, Tapanainen JS, Toppari J, Heikinheimo M. Developmental expression and spermatogenic stage specificity of transcription factors GATA-1 and GATA-4 and their cofactors FOG-1 and FOG-2 in the mouse testis. Eur J Endocrinol. 2002;147: 397–406. 12213678 10.1530/eje.0.1470397

[44] Jimenez-Severiano H, Mussard ML, Fitzpatrick LA, D'Occhio MJ, Ford JJ, Lunstra DD, et al Testicular development of Zebu bulls after chronic treatment with a gonadotropin-releasing hormone agonist. J Anim Sci. 2005;83: 2111–2122. 16100066 10.2527/2005.8392111x

[45] McClusky LM, Patrick S, Barnhoorn IE, van Dyk JC, de Jager C, Bornman MS. Immunohistochemical study of nuclear changes associated with male germ cell death and spermiogenesis. J Mol Histol. 2009;40: 287–299. 10.1007/s10735-009-9240-3 19924546

[46] Scudamore CL, Hodgson HK, Patterson L, Macdonald A, Brown F, Smith KC. The effect of post-mortem delay on immunohistochemical labelling-a short review. Comp Clin Path. 2011;20: 95–101.

[47] Karadzic R, Ilic G, Antovic A, Banovic LK. Autolytic ultrastructural changes in rat and human hepatocytes. Rom J Leg Med. 2010;18: 247–252.

[48] Tomita Y, Nihira M, Ohno Y, Sato S. Ultrastructural changes during in situ early postmortem autolysis in kidney, pancreas, liver, heart and skeletal muscle of rats. Leg Med. 2004;6: 25–31. 15177070 10.1016/j.legalmed.2003.09.001

